# Co-Expression of the Epstein-Barr Virus-Encoded Latent Membrane Proteins and the Pathogenesis of Classic Hodgkin Lymphoma

**DOI:** 10.3390/cancers10090285

**Published:** 2018-08-24

**Authors:** Katerina Vrzalikova, Maha Ibrahim, Eszter Nagy, Martina Vockerodt, Tracey Perry, Wenbin Wei, Ciaran Woodman, Paul Murray

**Affiliations:** 1 Institute of Cancer and Genomic Sciences, University of Birmingham, Birmingham B15 2TT, UK; ibrahim.maha@gmail.com (M.I.); E.Nagy@bham.ac.uk (E.N.); mvocker@gwdg.de (M.V.); PerryTA@adf.bham.ac.uk (T.P.); W.Wei@bham.ac.uk (W.W.); p.g.murray@bham.ac.uk (P.M.); 2 Institute of Anatomy and Cell Biology, Georg-August University of Göttingen, 37099 Göttingen, Germany; 3 Sheffield Institute of Translational Neuroscience, University of Sheffield, Sheffield S102HQ, UK; 4 Department of Clinical and Molecular Pathology, Institute of Molecular and Translational Medicine, Faculty of Medicine and Dentistry, Palacky University, 77515 Olomouc, Czech Republic

**Keywords:** EBV, LMP1, LMP2A, EBNA1, Hodgkin lymphoma, germinal centre

## Abstract

The Epstein-Barr virus (EBV) is present in the tumour cells of a subset of patients with classic Hodgkin lymphoma (cHL), yet the contribution of the virus to the pathogenesis of these tumours remains only poorly understood. The EBV genome in virus-associated cHL expresses a limited subset of genes, restricted to the non-coding Epstein-Barr virus-encoded RNAs (EBERs) and viral miRNA, as well as only three virus proteins; the Epstein-Barr virus nuclear antigen-1 (EBNA1), and the two latent membrane proteins, known as LMP1 and LMP2, the latter of which has two isoforms, LMP2A and LMP2B. LMP1 and LMP2A are of particular interest because they are co-expressed in tumour cells and can activate cellular signalling pathways, driving aberrant cellular transcription in infected B cells to promote lymphomagenesis. This article seeks to bring together the results of recent studies of the latent membrane proteins in different B cell systems, including experiments in animal models as well as a re-analysis of our own transcriptional data. In doing so, we summarise the potentially co-operative and antagonistic effects of the LMPs that are relevant to B cell lymphomagenesis.

## 1. Epstein-Barr Virus and the Pathogenesis of Classic Hodgkin Lymphoma

The Epstein-Barr virus (EBV) is associated with the pathogenesis of several types of B cell non-Hodgkin lymphoma (NHL; Burkitt lymphoma, diffuse large B cell lymphoma) as well as cases of classic Hodgkin lymphoma (cHL). These two major subgroups of B cell lymphoma are diagnosed on the basis of histopathological differences. cHL is characterised by the presence of rare usually single, malignant, so-called Hodgkin/Reed Sternberg (HRS) cells surrounded by non-neoplastic cell populations comprising T- and B-lymphocytes and other cell types. In contrast, in NHL, the malignant lymphocytes are the predominant population. HRS cells are B cells that have been arrested at the germinal centre (GC) or post-GC stages of B cell differentiation as evidenced by the detection of somatic mutations in immunoglobulin (Ig) V genes [1,2,3,4]. Around a quarter of cHL have so-called ‘crippling’ mutations in Ig V genes characterised by either nonsense mutations or deletions that destroy the ability of the gene to code for a functional Ig protein resulting in a cell that lacks a functional surface B cell receptor (BCR). GC B cells carrying such deleterious mutations are normally eliminated by apoptosis [3]. For this reason, it has been proposed that HRS cells originate from pre-apoptotic GC B cells that have been rescued from apoptosis by transforming events.

HRS cells also have a highly unusual transcriptional programme characterised by a striking downregulation of global B-cell lineage gene expression, including components of the BCR, as well as the aberrant co-expression of markers of other haematopoietic cell types including T-cells, NK-cells, and myeloid cells [5,6,7,8,9,10]. The loss of B cell identity is thought to be a consequence of the disruption of key B-cell associated transcription factor networks involving PAX5, early B-cell factor 1 (EBF1), and TCF3/E2A that regulate normal B cell development [11,12,13].

Aberrant transcription in HRS cells is also in part mediated by the constitutive activation of several key signaling pathways. HRS cells are characterized by constitutive activation the nuclear factor kappa B (NF-κB) transcription factors [14]. Inhibition of NF-κB signalling in cHL cell lines leads to enhanced apoptosis in response to withdrawal of growth factors and results in reduced tumour formation in mouse models [14]. HRS cells express CD30, CD40, TACI, BCMA, and RANK, all of which can induce the NF-κB activity [15,16,17,18], and there is evidence that the tumour microenvironment of cHL can contribute to the activation of these receptors [19,20]. Aberrant NF-κB activation can also be induced by mutations in key regulating genes. Thus, the c-REL subunit of NF-κB is overexpressed as a result of *REL* gene amplification [21,22,23]. NF-κB activity can also be increased by mutations of the genes encoding the IκB inhibitor proteins—IκB alpha and IκB epsilon—which normally act to inactivate NF-κB in the cytoplasm [24,25,26,27,28]. The non-canonical NF-κB pathway is also important for the survival of HRS cells, an effect mediated through RelB [29,30]. Mutations in *TNFAIP3/A20*, a negative regulator of NF-κB signaling, are also common in HRS cells [31].

JAK/STAT signalling also contributes to the proliferation and survival of HRS cells. Cytokines which can induce JAK/STAT activation are produced either by the HRS cells or by cells of the tumour microenvironment. This in turn leads to increased levels of phosphorylated forms of STAT3, STAT5, and STAT6 [32,33,34]. JAK/STAT signalling can also be aberrantly activated as a consequence of genetic changes that include *JAK2* amplifications or inactivating mutations in the negative regulators of STAT signaling, *SOCS1* and *PTPN1/PTPB1* [35,36,37].

The Epstein-Barr virus (EBV) is present in HRS cells in a subset of cases of cHL, but the fraction of positive cases is highly variable and dependent upon factors such as age, gender, histological subtype, ethnicity, and geographical locale [38,39,40]. EBV rates are high in cHL patients from less developed countries, but are lower in more developed Western populations, for example occurring at an incidence of between 20% and 50% in North American and European cHL patients [41,42]. As with other EBV-associated malignancies, the viral genomes are monoclonal in HRS cells, indicating that clonal expansion of the malignant cells occurred after EBV infection of a single infected progenitor B cell [43]. Moreover, EBV infection of HRS cells was shown to persist throughout the course of disease and to be present at multiple sites of disease, suggesting that EBV provides an important growth advantage to the HRS cell [44]. The importance of EBV in the pathogenesis of cHL is underscored by the observation that cHL cases with ‘crippling’ mutations are almost always EBV-positive and by the finding that EBV is capable of immortalizing GC B cells lacking a functional BCR [45,46,47,48].

In keeping with other forms of EBV-associated B cell lymphoma, a defect of immune surveillance is suspected to be an important factor in the pathogenesis of EBV-positive cHL. Thus, there is an increased frequency of EBV-associated cHL following solid organ transplantation [49,50] and allogeneic haematopoietic stem cell transplant [51]. EBV-positive cHL is also the most frequent non-acquired immunodeficiency syndrome (AIDS) defining cancer diagnosed in HIV infected individuals. However, EBV-positive cHL also occurs in apparently immunocompetent individuals. In older people, this may be due to senescence of EBV-specific immunity, paralleling the increased incidence of EBV-positive DLBCL associated with advancing age [52]. However, the specific nature of the defects in EBV-specific immunity that predispose individuals to an increased risk of EBV-positive cHL have yet to be identified.

## 2. The Role of EBV Latent Membrane Proteins in Viral Persistence

A detailed understanding of the transforming properties of EBV in B cells have mainly utilized a well-established in vitro system in which B cell proliferation and survival are induced by the coordinated action of all the EBV latent genes (a pattern of EBV gene expression known as latency III). The end result is the generation of continuously proliferating B cell lines known as a lymphoblastoid cell lines (LCL). Several EBV latent genes, including EBNA2 and LMP1, as well as the EBNA3A and EBNA3C genes have been shown to be essential for the in vitro transformation of B cells in this model [53,54,55]. EBNA1 is also considered essential since it is required for the maintenance of EBV infection, having key functions in virus genome replication and in the segregation of viral genomes to daughter cells during cell division [56,57]. EBNA1 is also a transcriptional regulator of both viral and cellular genes [58,59,60,61,62]. However, in contrast to the LCL model, the majority of EBV-associated cancers display much more restricted patterns of virus gene expression, in which EBNA2, EBNA3A, and EBNA3C are usually not expressed [63]. The EBV genome in HRS cells, for example, expresses a restricted pattern of virus latency, known as latency II, characterised by the presence of EBNA1, the two latent membrane proteins LMP1 and LMP2, the Epstein Barr encoded RNAs (EBERs), and the viral miRNA [64,65,66]. The focus of this article is the contribution of the EBV latent membrane proteins to the pathogenesis of cHL. We first provide a summary of EBV infection in the normal host before considering the contribution of the viral latent membrane proteins to the development of cHL.

EBV persists in the memory B cells of normal asymptomatic virus carriers [67]. Virus proteins are not expressed in most EBV-infected memory B cells, a viral gene expression pattern known as latency 0 [68]. While EBNA1 is not expressed in the quiescent EBV-infected memory B cell pool, it is required when these cells undergo proliferation. This pattern of virus gene expression is referred to as latency I [68]. In asymptomatic carriers, the latent membrane proteins LMP1 and LMP2A have key roles in the differentiation of normal EBV-infected GC cells to memory B cells and are therefore also critical for virus persistence. In the so-called ‘germinal centre model’ of virus persistence, LMP1, a constitutively active CD40 receptor homologue [69,70], and LMP2, a BCR mimic [71,72], are thought to provide the signals necessary for the survival and antigen-independent differentiation of EBV-infected GC B cells into memory B cells. EBV-infected GC B cells can also differentiate into plasma cells [73], but there is evidence that LMP1 can suppress this process, potentially to ensure that the majority of EBV-infected GC B cells are directed into the memory B cell compartment [74]. Clearly, a small subset of EBV-infected B cells does indeed eventually progress to become plasma cells and this process could be driven in part by LMP2A [75,76,77]. The net result is that some EBV-infected plasma cells arrive in the tonsils, where they complete the viral replicative cycle and release new virions into saliva for transmission to other human hosts.

## 3. EBV Latent Membrane Proteins and the Pathogenesis of Classic Hodgkin Lymphoma

Although LMP1 is seemingly important for normal virus persistence, it is also implicated in cell transformation. LMP1 behaves as a classic oncogene in vitro [78] and is indispensable for the immortalisation of B cells by EBV in the LCL model [54]. As a constitutively active variant of the CD40 receptor, LMP1 induces many of the cell signalling pathways described above that are aberrantly activated in HRS cells, including the NF-κB, JAK/STAT, AP-1, and (PI3K)/AKT pathways [79,80,81,82,83,84,85,86]. Activation of these signalling pathways by LMP1 contributes to the aberrant transcriptional programme of HRS cells. Thus, using primary human GC B cells as a model, we showed that LMP1 alone could recapitulate almost one-quarter of the transcriptional changes observed in cHL cells, including the characteristic loss of B cell identity [87]. LMP1 is also important for B cell survival and could promote the survival of EBV-infected BCR-negative HRS progenitors also through its ability to increase the expression of anti-apoptotic molecules, including BCL2 and MCL1 [88,89,90].

LMP2 exists as two isoforms, LMP2A and LMP2B, which share 8 common coding exons but have different 5′ exons. LMP2A functions as a BCR mimic, allowing B cell development in the absence of normal BCR signalling [71,72,91]. LMP2A activates RAS/PI3K/AKT signalling which is important for B cell survival [92], as well as the mTOR pathway [93]. LMP2A is not essential for B cell proliferation or for the routine in vitro immortlisation of B cells by EBV [94,95]. However, it is critical for the EBV-induced immortalisation of BCR-negative GC B cells [46,47,48,96]. Even though the BCR signalling machinery is not functional in HRS cells, LMP2A expression in different B cells systems can induce many of the cellular transcriptional changes characteristic of HRS cells [97,98].

**Figure 1:**
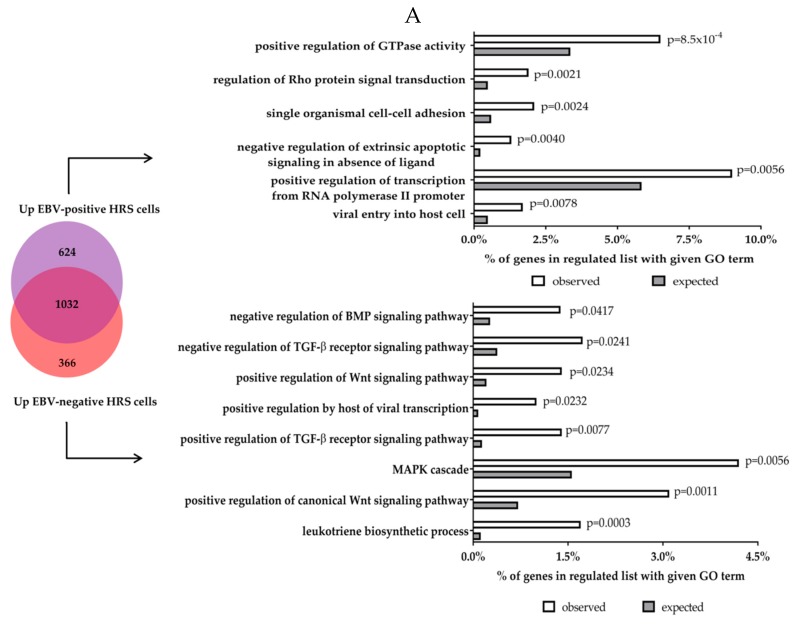

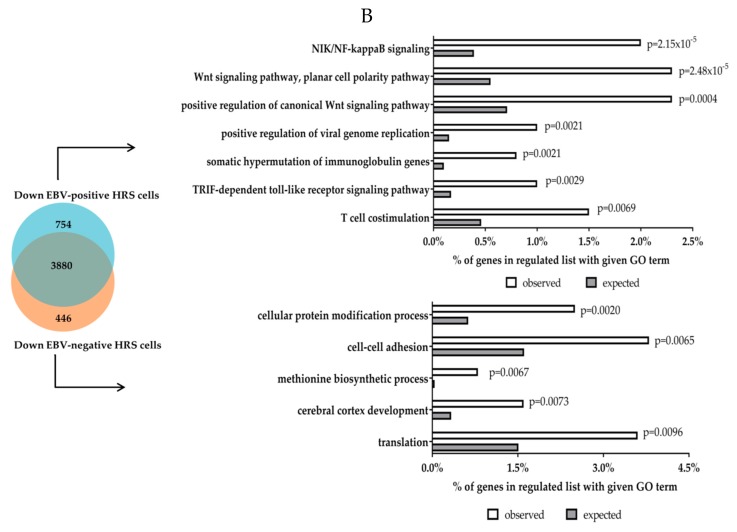
Gene ontology (GO) analysis of genes upregulated (**A**) and downregulated (**B**) in either EBV-positive or EBV-negative HRS cells. Gene lists were derived from our reanalysis of genes differentially expressed in either EBV-positive or EBV-negative HRS cells compared to microdissected normal GC. Gene set enrichement was performed using DAVID 6.8 (Frederick, MD, USA) [99,100].

Our re-analysis of published data showing that the transcriptional changes present in EBV-positive and EBV-negative HRS cells are very similar (Figure 1) supports the notion that while the pathogenic mechanisms occurring in EBV-positive and EBV-negative cHL may be different they mostly converge on the same transcriptional end-points. The current model is one in which EBV infection can functionally substitute for cellular genetic changes. In support of this it has been shown that there is an inverse relationship between EBV infection and *TNFAIP3* mutations [31]. This possibility is further supported by a recent study of the global genomic landscape of cHL showing that cellular mutations are more common in EBV-negative compared to EBV-positive HRS cells [9]. However, it is noteworthy that in our reanalysis we also observed subsets of genes that were up- or down-regulated only in EBV-positive or EBV-negative HRS cells, but not in both. A gene ontology (GO) analysis of these gene subsets revealed the potential existence of distinct pathogenic mechanisms in the virus-positive and -negative forms of cHL. We found that genes up-regulated only in EBV-positive HRS cells were enriched for genes associated with GTPase activity and Rho signaling as well as viral entry to host cells. In contrast, genes up-regulated only in EBV-negative tumours were enriched for genes with functions in the positive regulation of WNT signalling, whereas genes associated with WNT signaling were down-regulated in EBV-positive HRS cells, suggesting that WNT signaling might be important only in EBV negative tumours. We also found that genes down-regulated only in EBV-positive HRS cells included genes with functions in TLR signalling and T cell co-stimulation, suggesting that evasion of inate and adaptive immunity could be more important in EBV-positive cHL.

## 4. Effects of the Latent Membrane Proteins on the Phenotype of B Cells in Mouse Models

All the reported mouse models of B cell lymphoma have so far not been able to recapitulate the cHL phenotype and therefore are only representative to varying degrees of different NHL. Nonetheless, they have provided useful insights into the pathogenic consequences of the expression of the LMPs in the B cell system that are likely to have relevance to our understanding of the role of these viral genes in cHL. In particular, they have enabled an exploration of the effects of co-expression of the LMPs on a B cell background. Keeping in mind these important caveats we now present a summary of the effects of LMP1 and LMP2A in mouse models of B cell malignancy.

CD40 and LMP1 signaling have been compared in several mouse models. In CD40-deficient mice, LMP1 expressed under the control of the Ig promoter/enhancer restored CD40 signals to induce extrafollicular B cell activation, differentiation and Ig class switching [101]. However, unlike CD40, LMP1 prevented GC formation in these animals [101]. Several studies have utilized artificial CD40-LMP1 chimeras consisting of the extracellular and transmembrane domains of CD40 fused to the cytoplasmic tail of LMP1. In these chimeras LMP1 signalling is activated through ligation of CD40L to a normal CD40 extracellular domain. Studies using these chimeras show that LMP1 signaling is intrinsically similar to CD40 signaling [102,103]. Thus, the CD40-LMP1 chimeric mice have normal lymphocytes, and immunization resulted in apparently normal antibody response including isotype switching, affinity maturation, and GC formation. However, in one of these studies, unimmunized mice did show some abnormalities, including expanded immature and GC B cells, with production of autoantibodies [102]. In contrast, B cells of transgenic mice expressing an LMP1-CD40 chimera (in which the C-terminus of LMP1 is replaced by the C-terminus of CD40 and so CD40 signaling is now constitutive) displayed an activated phenotype, protracted survival, and increased proliferation [104]. Furthermore, these mice developed B cell lymphomas at an advanced age (>12 months) [104], which is compatible with the late onset of lymphomas developing in LMP1 transgenic mice [105]. These data indicate that it is the constitutive nature of the LMP1 signal that promotes oncogenesis.

In contrast to LMP1, mice expressing LMP2A under the control of the Ig heavy chain gene promoter/enhancer have phenotypically normal BCR-positive B cells and do not show an increased propensity for lymphoma development [72,91]. This model has also been used extensively to show several important facets of LMP2A function, including its ability to provide a BCR-like survival signal and to increase antibody production through expansion of the activated B cell pool as well as increasing the number of plasma cells [72,91,106]. Minimatani et al. has also reported the effects of LMP2A expression in a similar model; this time LMP2A was targeted to CD19-positive mouse B cells and showed that LMP2A enhanced B cell proliferation in vivo in response to BCR activation, and increased the number of antibody secreting cells [75]. LMP2A also augmented extrafollicular B cell differentiation and led to reduced GC formation following immunisation [75].

The transgenic mouse models so far described have studied the effects of LMP1 or LMP2A when these genes were expressed in most, if not all, of the B cells in these animals, including cells representing the pre-GC stages of B cell differentiation. As such, these models are unable to recapitulate the probable restricted expression of the latent membrane proteins in GC-like B cells of asymptomatically infected individuals. In an attempt to address this issue several groups have expressed LMP1 and/or LMP2A specifically in GC B cells; although an improvement on the previous models of expression in all B cells, these models also cannot take account of the likelihood that EBV-positive cHL derives from a distinct post-GC stage of B cell differentiation/activation [107].

Minimatani et al. expressed LMP2A specifically in the GC B cells of mice (under control of the GC-specific AICDA promoter) [75]. They found that LMP2A expression in GC B cells resulted in impaired antigen-specific antibody responses as a consequence of the preferential selection of low affinity B cells, but in contrast to its expression in CD19-positive B cells, LMP2A expression in GC B cells had no effect on the size or morphology of GC [75].

In contrast to the pathologies observed in mice when LMP1 and LMP2 are expressed separately under the control of the Ig heavy chain gene promoter/enhancer, Vrazo et al. showed that B cells are normal when LMP1 and LMP2A are co-expressed in this model [108]. Furthermore, LMP2A was shown to dampen LMP1-induced hyperproliferation in response to mitogen treatment and restored normal GC formation, allowing LMP1-expressing cells to populate the GC. LMP1/2A double transgenic B cells also differentiated normally into plasma cells at a frequency similar to wild-type controls. Moreover, LMP2A decreased the expression of TRAF2, a critical component of LMP1-mediated induction of NF-κB signalling, suggesting a possible mechanism to explain the counteracting effects of LMP2A on LMP1 function in this model [108]. These observations are consistent with the previous finding that LMP2A expression reduces signal transduction in a B cell lymphoma cell line (BJAB) transfected with LMP1 and LMP2A [109], but contradict the results of Guasparri et al., who showed that the knockdown of either LMP1 or LMP2A in EBV-infected B cell lymphoma cell lines significantly reduced NF-ĸB activity and increased their susceptibility to apoptosis, an effect that was attributed to the up-regulation of TRAF2 by LMP2A [110]. Thus, TRAF2 seems to be a critical player in mediating the effects of LMP2A on LMP1 functions.

Further evidence for potential synergy between LMP1 and LMP2A was provided by a transgenic mouse model of conditional co-expression of LMP1 and LMP2A in GC B cells driven from the AICDA promoter [76]. Immunosuppression induced by combined treatment of these animals with anti-T cell and anti-NK cell antibodies caused rapidly fatal lymphoproliferative disease, death occurring in 50% of animals within eight days [76]. In contrast, the survival of single LMP2A- or LMP1-expressing GC mice was unaffected up to two weeks after administration of anti-T and anti-NK antibodies. LMP1/2A co-expression in this model also induced GC B cell and plasmablast differentiation to a greater extent than was observed in mice expressing only LMP2A in GC B cells, effects that were accompanied by the over-expression of major regulators of plasma cell differentiation (PRDM1, XBP1) and the down-regulation of genes involved in maintaining the GC phenotype (e.g., BCL6) [76]. Furthermore, LMP1/LMP2A co-expression in GC B cells did not cause lymphoproliferative disease in immunocompetent mice, providing further supportive evidence that immune suppression/evasion is a key component of the pathogenesis of virus-driven lymphomas. Zhang et al. also showed that the immune system is important in controlling the outgrowth of LMP1-positive CD19-positive B cells in transgenic mice [111].

Wirtz et al. co-expressed LMP1 and LMP2A in the CD19-positive B cell compartment of immunocompetent mice [112]. While combined LMP1/LMP2A expression in all B cells was rapidly fatal, the expression of these virus proteins in a minority of the B cell pool led to the expansion of the LMP1/LMP2A expressing B cell population, which was followed by their recognition by T cells and eventual disappearance [112]. However, when these experiments were performed following T cell depletion or in animals deficient in perforin, a protein important for target cell killing by CD8 T cells, the result was fatal LMP1/LMP2A-driven lymphoproliferative disease. The same effect was observed when these experiments were performed in immunised mice expressing LMP1/LMP2A specifically in GC B cells but was not observed in mice expressing either LMP1 or LMP2A alone in GC B cells. Of note was the observation that LMP1, and to a greater extent LMP1 and LMP2A when expressed together, reduced the number of GC B cells, whereas LMP2A expression alone in GC B cells had no effect on GC B cell numbers [112].

Shair et al. studied the transcriptional effects of the single expression of LMP1 or LMP2A, or their combined expression in B cells under the control of the Ig promoter/enhancer [113]. They found that 70% of the cellular genes differentially expressed in mouse B cells co-expressing LMP1 and LMP2A were also differentially expressed when LMP1 or LMP2A were expressed alone; the remaining 30% of genes were uniquely regulated by either LMP1 or LMP2A. Comparison of the transcriptional effects of LMP1 in transgenic mouse B cells with those observed in human GC B cells transfected with LMP1, in cHL cell lines or regulated by LMP1 in Burkitt lymphoma cell lines and LCL, revealed only a modest overlap. These observations could reflect differences in the effects of LMP1 in human versus mouse B cells, or in transformed compared with normal B cells. It is noteworthy that this study also showed that LMP1 and LMP2A both enhanced the viability of transgenic B cells when expressed singly, yet surprisingly, B cells co-expressing LMP1 and LMP2A had a viability that was similar to littermate controls [113].

Recently, it was shown that co-injected CD40L-expressing T cells were required for the ability of an LMP1-deleted EBV to induce Lat III-like lymphomas in a cord-blood humanised mouse model [114]. Hence, it appears that signals from the tumour microenvironment can partially substitute for the lymphomagenic effects of LMP1. Furthermore, a recent study from the same group has shown that infection with a recombinant EBV lacking LMP2A results in the occurrence of lymphomas at a rate that is similar to that observed in wild-type-virus infected animals, although lymphoma development was somewhat delayed [77]. Tumours still occurred, albeit at a reduced frequency, when cells were infected with an EBV lacking both LMP1 and LMP2A, pointing to the importance of other latent genes, most probably EBNA2, EBNA3A, and EBNA3C. It should also be noted that the LMP1/LMP2A deleted virus used in these experiments was profoundly defective for the in vitro transformation of B cells further underscoring the importance of the tumour microenvironment in the pathogenesis of EBV-associated lymphomas [77].

## 5. Transcriptional Effects of LMP1 and LMP2 in Primary Human Germinal Centre B Cells

**Figure 2:**
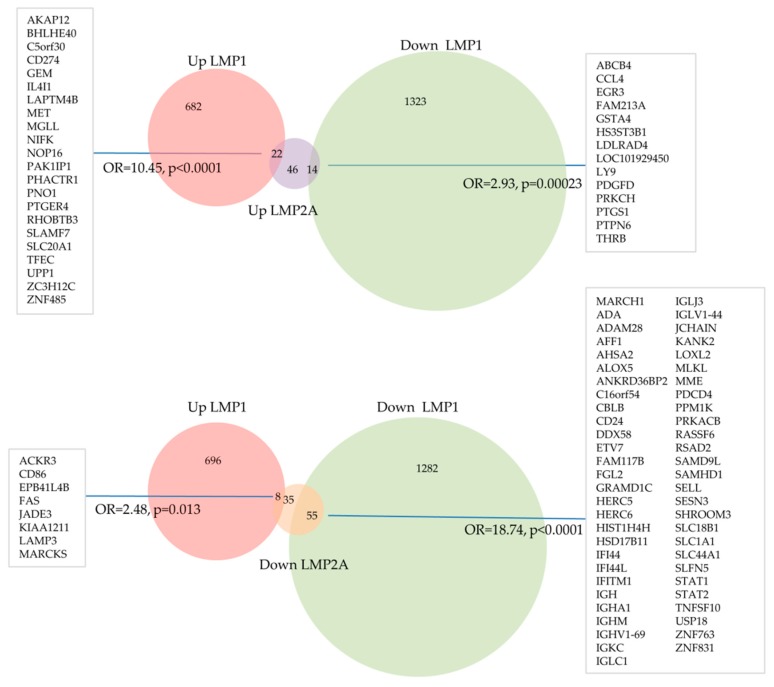
Overlap between LMP1- and LMP2A-regulated genes in primary human GC B cells. Genes differentially expressed following transfection of normal GC B cells with either LMP1 or LMP2A identified in our reanalysis of existing published data. Statistical significance of overlap between gene sets was determined using Chi-square test.

**Figure 3:**
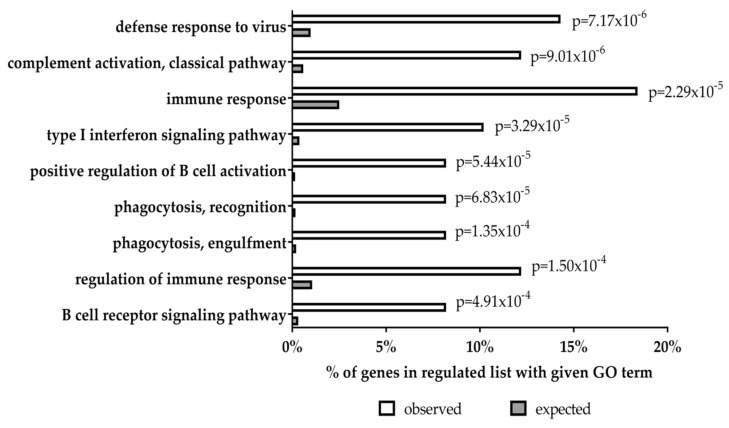
GO analysis of genes downregulated by both LMP1 and LMP2A in GC B cells. Gene lists were derived from our reanalysis of genes differentially expressed in either LMP1 or LMP2A expressing GC B cells. Gene set enrichment was performed using DAVID 6.8 [99,100].

We have previously used the transfection of primary human GC B cells to study the impact of the EBV latent genes on the cellular transcriptome of these cells [74,87,98]. A re-analysis of our previous microarray datasets has revealed that 704 unique genes were up-regulated and 1337 unique genes were down-regulated following LMP1 expression in primary human GC B cells. 82 genes were up-regulated and 98 genes were down-regulated by LMP2A in the same cell background. Of these, 22 and 55 genes were also up- and down- regulated, respectively, by LMP1. This represents a highly significant overlap (odds ratio (OR) = 10.45, *p* < 0.0001, and OR = 18.74, *p* < 0.0001, respectively; Figure 2) which could explain some of the synergistic/additive effects of LMP1 and LMP2A observed in mouse B cells described above. In particular, we found a striking overlap of genes down-regulated by both LMP1 and LMP2A that are involved in the regulation of host immune responses, including for example, key components of type I interferon responses such as *STAT1*, *STAT2*, *IFITM1*, and *IFI44L* (Figure 3). This presumably underscores the importance to the virus in disabling this key anti-viral defense pathway. However, we also found that genes up-regulated by LMP1 were significantly enriched among genes down-regulated by LMP2A in GC B cells (OR = 2.48, *p* = 0.013). Moreover, genes down-regulated by LMP1 were significantly enriched among genes up-regulated by LMP2A (OR = 2.93, *p* = 0.00023). Genes regulated in opposite directions included *CD86* and *FAS* which were up-regulated by LMP1 but down-regulated by LMP2A. This could be relevant because it has been observed that cross-linking of CD80 and CD86 in EBV-infected lymphoblastoid cell lines results in apoptosis [115]. This effect was a consequence of the induction of FAS and FASL expression by CD80 and CD86 stimulation. Therefore, LMP2A could be important for counteracting the potentially apoptotic effects of EBV infection of B cells.

**Figure 4:**
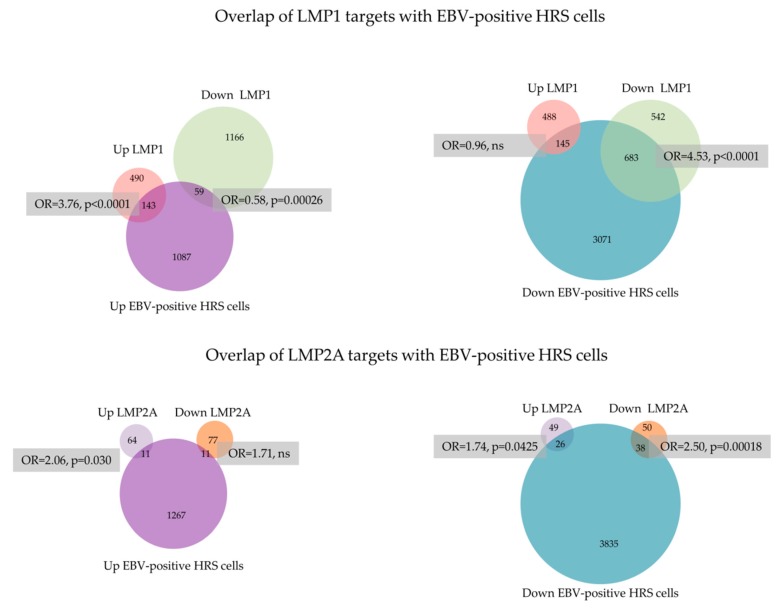
Overlap of LMP1 and LMP2A targets with EBV-positive HRS cells. Genes differentially expressed following transfection of normal GC B cells with either LMP1 or LMP2A were compared with those differentially expressed in EBV positive HRS cells. Statistical significance of overlap between gene sets was determined using Chi-square test.

A re-analysis of published gene expression datasets from B cell lymphomas enabled us to explore the potential contribution of the overlapping and counteracting transcriptional programmes of LMP1 and LMP2A to cHL. To do this, we identified the genes differentially expressed in EBV-positive HRS cells compared with normal GC [116]. Confirming previous reports, we found that LMP1 targets were significantly enriched among genes concordantly regulated in EBV-positive cHL. In contrast, the effects of LMP2A were less clear cut; while genes regulated by LMP2A were significantly enriched among genes concordantly regulated in HRS cells, we also observed an enrichment of genes up-regulated by LMP2A among genes down-regulated in HRS cells (Figure 4).

## 6. Conclusions

A number of studies have attempted to unravel the phenotypic effects of the latent membrane proteins either when expressed singly, or in combination, using different in vitro systems and animal models. However, in many cases, these studies have yielded conflicting results, producing a confusing picture of the effects of the latent membrane proteins in B cells. In spite of these differences, some general conclusions can be drawn. First, LMP1 has been shown to consistently transform B cells in different experimental systems. Furthermore, a constitutive CD40 signal delivered by an LMP1/CD40 chimera is also transforming. In contrast, CD40/LMP1 chimera in which LMP1 signaling is regulated by a physiological CD40L stimulus is not transforming. These data indicate it is the constitutive nature of the LMP1 signal that is transforming. Second, LMP2A can functionally replace a BCR signal in B cells. Although not yet shown, this could be important for the survival of BCR negative HRS progenitors. Third, co-expression of LMP1 and LMP2A in B cells of immunocompetent mice has been shown to reverse the abnormalities induced when these latent genes are expressed separately. However, when LMP1 and LMP2A are co-expressed in a background of immunodeficiency the result is a fatal lymphoproliferative disease. These data indicate that the immune system can more efficiently delete cells co-expressing both latent membrane genes. There are also a number of unanswered questions. First, what are the precise roles of the latent membrane proteins during the establishment and maintenance of persistent infection in the B cells of normal asymptomatic carriers, and how do temporal changes in the expression of LMP1 and LMP2A impact on GC B cell differentiation and subsequent memory B cell or plasma cell development? Second, are LMP1 and LMP2A co-expressed in primary EBV-associated cancers and how do they regulate cellular functions either together or separately in different lymphoma subtypes derived from distinct developmental stages. This question is relevant also to epithelial cancers, including the subset of nasopharyngeal carcinomas in which LMP1 and LMP2A are known to be expressed together. There is already evidence of potential synergy in epithelial carcinogenesis. For example, LMP2A can augment LMP1 signalling in epithelial cells by increasing LMP1’s half-life [117] and LMP1 and LMP2A co-expression driven by the keratin 14 promoter can accelerate carcinogenesis in transgenic mice [118]. Third, how are the effects of LMP1 and LMP2A modulated by other latent genes, e.g., EBNA1, the viral miRNA and the EBERs? Fourth, how do normal and malignant tissue micro-environments regulate the expression and functions of the latent genes, and in turn how do the EBV latent genes modify tissue microenvironments to promote, or even potentially suppress, tumourigenesis? Fifth, it is not known what molecular events in cooperation with viral genes dictate the eventual tumour type (cHL, NHL, Burkitt lymphoma, diffuse large B cell lymphoma) that results from virus driven transformation events. Finally, we still do not understand how these virus proteins contribute to established malignancies; this is particularly relevant in the case of LMP2A, a BCR homologue that is expressed in the malignant cells of cHL despite the fact that these cells lack the BCR signalling molecules upon which LMP2A’s BCR-like effects depend. We envision that these questions will be important, not only for our understanding of EBV and its role in oncogenesis, but also for the development of therapeutic strategies that target key viral functions in the malignant cell.
